# Cellular functions of the ClpP protease impacting bacterial virulence

**DOI:** 10.3389/fmolb.2022.1054408

**Published:** 2022-12-01

**Authors:** Mazen E. Aljghami, Marim M. Barghash, Emily Majaesic, Vaibhav Bhandari, Walid A. Houry

**Affiliations:** ^1^ Department of Biochemistry, University of Toronto, Toronto, ON, Canada; ^2^ Department of Chemistry, University of Toronto, Toronto, ON, Canada

**Keywords:** ClpP protease, virulence, pathogenesis, ATP-dependent proteases, substrates

## Abstract

Proteostasis mechanisms significantly contribute to the sculpting of the proteomes of all living organisms. ClpXP is a central AAA+ chaperone-protease complex present in both prokaryotes and eukaryotes that facilitates the unfolding and subsequent degradation of target substrates. ClpX is a hexameric unfoldase ATPase, while ClpP is a tetradecameric serine protease. Substrates of ClpXP belong to many cellular pathways such as DNA damage response, metabolism, and transcriptional regulation. Crucially, disruption of this proteolytic complex in microbes has been shown to impact the virulence and infectivity of various human pathogenic bacteria. Loss of ClpXP impacts stress responses, biofilm formation, and virulence effector protein production, leading to decreased pathogenicity in cell and animal infection models. Here, we provide an overview of the multiple critical functions of ClpXP and its substrates that modulate bacterial virulence with examples from several important human pathogens.

## Introduction and background

### Caseinolytic protease is a drug target for antimicrobial development

Antimicrobial resistance to common treatments continues to be one of the largest threats to global public health. Therefore, new antibiotics with new modes of action are in great demand. Molecular chaperones and proteases play a critical role in maintaining cellular protein homeostasis (proteostasis). Dysregulation of these proteostasis mechanisms has been shown to disrupt major cellular pathways and is lethal for many organisms (Ingmer and Brondsted, 2009; Frees et al., 2013; Bhandari et al., 2018). Due to their importance in proteostasis and the lack of preexisting resistance associated with them, chaperones and proteases have recently emerged as promising targets for the development of novel antimicrobial compounds (Raju et al., 2012a; Brӧtz-Oesterhelt and Sass, 2014).

Here, we discuss one such candidate for antibiotic targeting, the caseinolytic protease (ClpP) that is conserved across many kingdoms of life. ClpP is a serine protease that functions with cognate unfoldase ATPases. In bacteria, it acts as one of the major ATP-dependent cellular proteases along with Lon, HslUV, and FtsH (Culp and Wright, 2017). ClpP functions in the removal of damaged and unwanted proteins and also in the degradation of regulatory proteins. Additionally, ClpP is integral to many important cellular pathways across different bacterial species. Its substrates include proteins involved in cell cycle regulation, stress tolerance, virulence factor production, biofilm formation, antibiotic tolerance, and metabolism (Gottesman, 2019; Kirsch et al., 2021). Though it plays important roles across a variety of bacterial pathogens, its influence varies between different species. The role of ClpP in the pathogenesis of select species is explored below along with a discussion on factors that determine its substrates.

### Overview of caseinolytic protease structure

**FIGURE 1:**
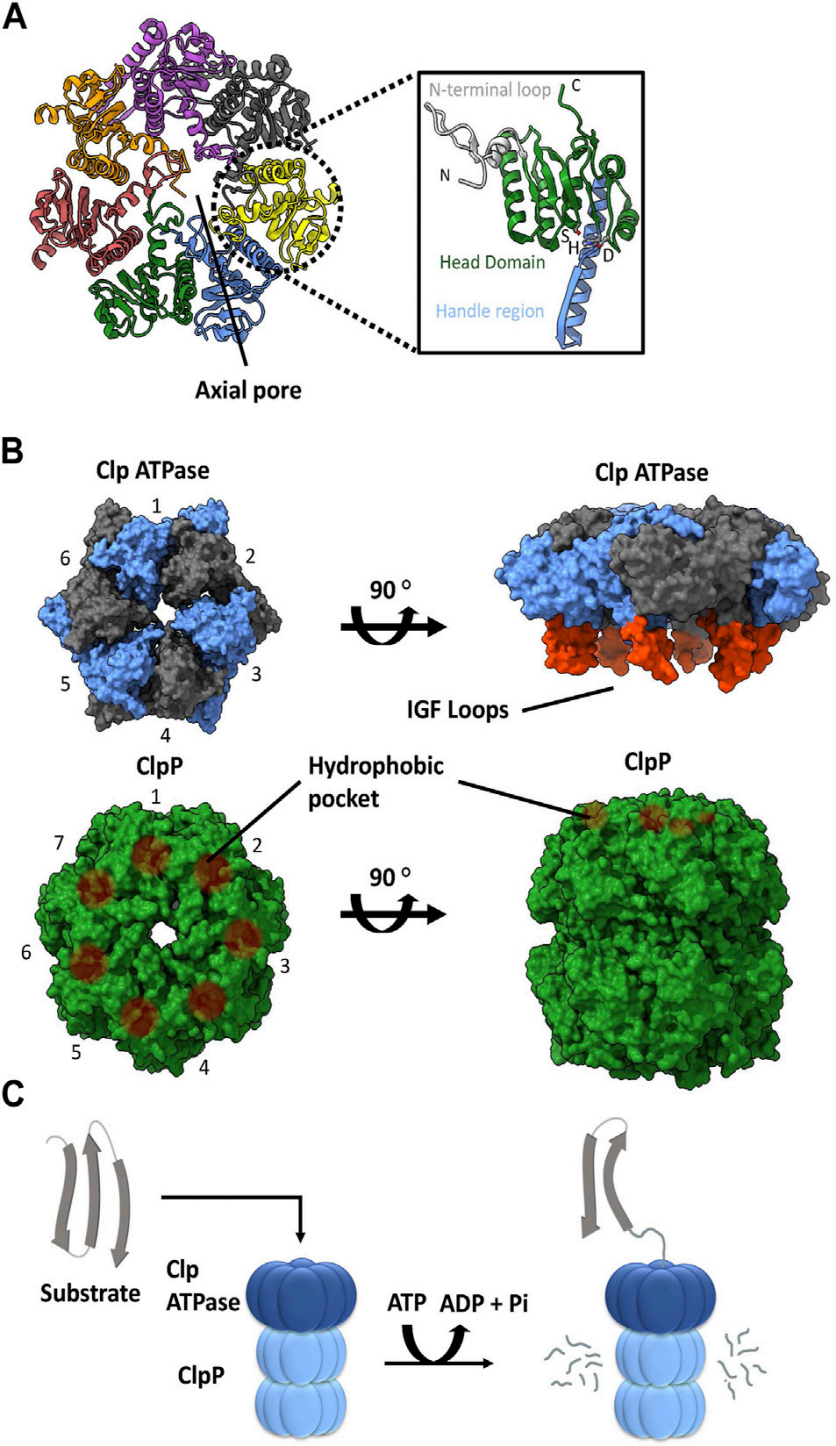
ClpP and Clp ATPases general architecture. **(A)** Top view of the *E. coli* ClpP cylinder (PDB ID: 1YG6). In the illustration on the right, the N-terminal loop (silver), head domain (green), handle region (blue) and catalytic triad (S, H, D) of the ClpP monomer are indicated (PDB ID 1YG6). **(B)** The association of a Clp ATPase hexamer (PDB ID 6SFW) with a ClpP tetradecamer (PDB ID 6SFX). Clp ATPase subunits (blue and grey) with their corresponding IGF loops (orange) are shown. ClpP subunits (green) and their hydrophobic pockets are denoted as brown circles. **(C)** General schematic of the proteolytic degradation cycle mediated by ClpP with its cognate Clp ATPase. Substrates are unfolded by the Clp ATPase and then translocated in an ATP-dependent manner into the ClpP catalytic chamber for proteolysis.

ClpP is a self-compartmentalizing serine protease. It is composed of fourteen subunits that typically assemble into a stack of two heptameric rings to create a hollow barrel-like structure. Each protomer typically contains a Ser-His-Asp catalytic triad facing the interior of the complex, forming the catalytic chamber. A ClpP subunit is composed of three subdomains or regions: N-terminal loops, the head domain, and the handle region (Figure 1A). The N-terminal loops are also called the axial loops, which line the entrance of the axial pore of the tetradecameric complex and protrude from the apical surface of ClpP (Figure 1A). The pore lining is comprised of hydrophobic residues that are stabilized by interactions with the head domain, whereas the axial protrusion is comprised of hydrophilic or charged residues. The head domain forms the main body of the degradation chamber, while the handle region facilitates the interaction between the two heptameric rings by intercalation. When ClpP is not associated with its cognate ATPase, the N-terminal regions are typically disordered and partially block the entrance to the degradation chamber (Moreno-Cinos et al., 2019; Kahne and Darwin, 2021; Mabanglo and Houry, 2022). This ensures that cellular proteins are protected from uncontrolled proteolysis by the catalytic residues within the ClpP degradation chamber. On its own, the ClpP tetradecamer can degrade short peptides that are small enough to pass through the axial pores. Proteolysis of larger proteins requires the formation of a complex with a Clp ATPase chaperone (Figures 1B,C) (Liu et al., 2014).

### Clp ATPase chaperones

A variety of Clp ATPases belonging to the Hsp100 class of the AAA+ superfamily (ATPases associated with various cellular activities) have been identified. These include ClpA, ClpB, ClpC, ClpE, ClpL, and ClpX chaperones. They typically form a hexameric structure with a central pore (Figures 1B). They usually contain an N-terminal domain followed by one or more ATPase domains. As such, the Clp ATPases serve two functions: selection and binding of a substrate protein, followed by its unfolding and subsequent translocation into the ClpP catalytic chamber (Olivares et al., 2016) (Figure 1C).

With the exception of ClpB, all of the other Clp ATPases can associate with a ClpP. The ability to do so is dependent on the presence of ClpP recognition loops. These loops contain the IGF tripeptide motif in gamma-proteobacteria, but they can be MGF or LGF in other bacterial phyla (Amor et al., 2019). The 6 M/L/IGF loops of the Clp ATPases dock onto specific hydrophobic pockets on the surface of ClpP (Figures 1B). These hydrophobic pockets are located near the outer edge of the apical surface between the ClpP protomer subunits. The 6:7 Clp ATPase to ClpP symmetry mismatch has been a topic of debate for decades. However, recently three Cryo-EM structures of bacterial ClpXP were solved which show that ClpX docks in an offset manner from ClpP in a tilted position. This allows 6 of the 7 hydrophobic pockets in ClpP to interact with the IGF loops of ClpX, thereby leaving one hydrophobic pocket empty (Gatsogiannis et al., 2019; Fei et al., 2020b; Ripstein et al., 2020). Consequently, this 6:7 symmetry mismatch seems to be required for target substrate unfolding and translocation into the ClpP catalytic chamber (Figures 1C); however, the molecular basis and consequences of this symmetry mismatch on ClpXP activity is not clear.

**FIGURE 2:**
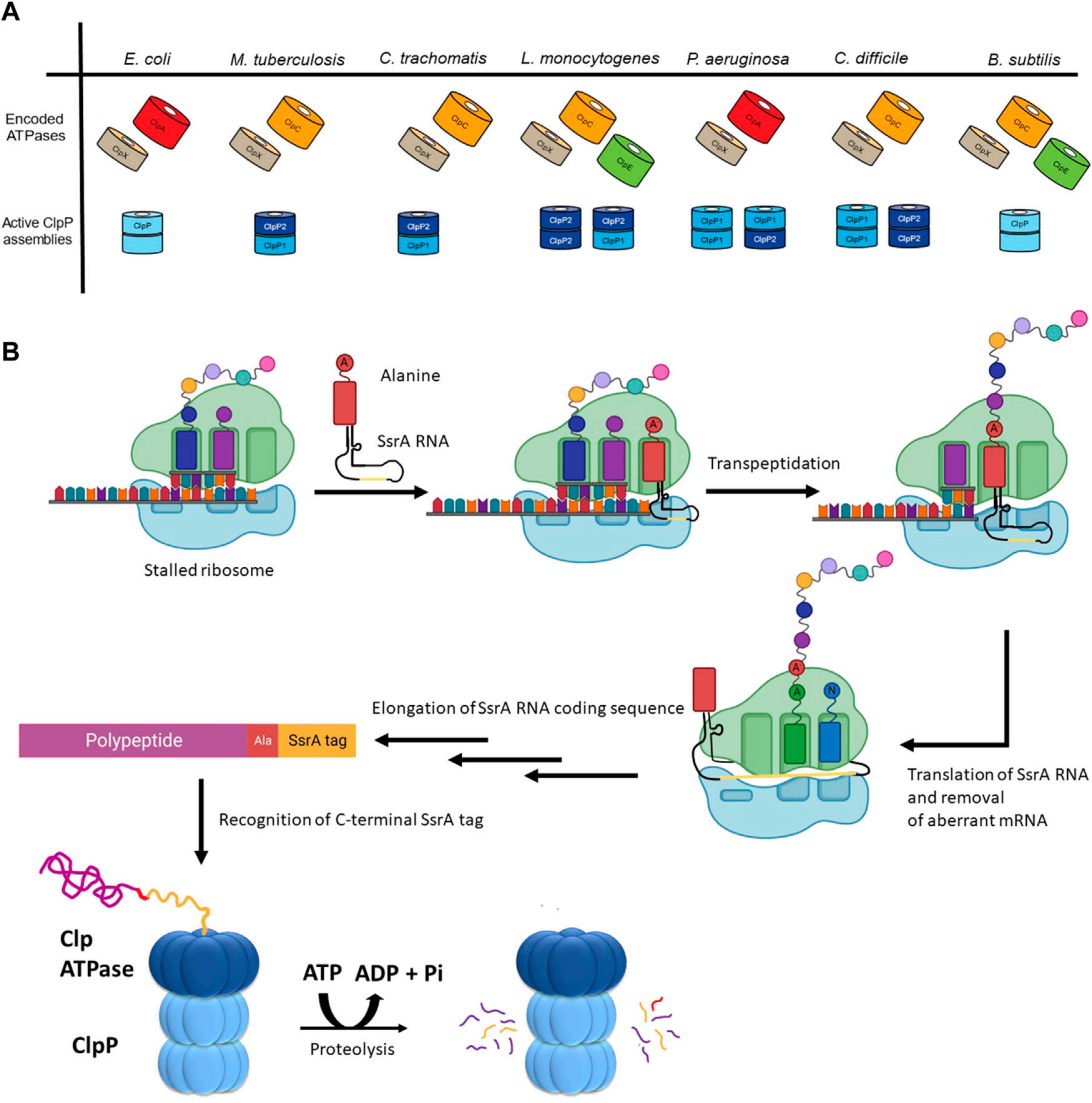
The diversity of ClpP substrate regulation. **(A)** The Clp ATPases and ClpPs of a select group of bacterial pathogens are shown. The tetradecameric organization for the active form(s) of the ClpP protease for each of these species is also shown. **(B)** Shown is the mechanism of SsrA tagging of nascent chains as means of targeting them for ClpP-mediated degradation. During translation of aberrant mRNAs lacking a stop codon, the ribosome stalls, resulting in the recruitment of a charged alanine-SsrA RNA (tmRNA) to the A site of the ribosome. Following transpeptidation of the charged alanine-residue to the nascent chain, the aberrant mRNA open reading frame is replaced with the RNA carried by the tmRNA, resulting in the translation of the SsrA RNA coding sequence (yellow). This C-terminal tag placed on the stalled nascent chains acts as a degron that is recognized by the Clp ATPase (e.g., ClpX or ClpA) enabling ClpP-mediated degradation.

The number and type of Clp ATPases found in different species varies even among closely related organisms (Figure 2A). However, some trends have been observed. ClpX is the most widespread ATPase and can be found in almost all bacteria. ClpA is generally found among the Gram-negative proteobacteria. ClpC is present in the firmicutes and actinobacteria groups, as well as in species of cyanobacteria, while the presence of ClpE is limited to some species of the firmicutes group (Kress et al., 2009).

### Diversity of caseinolytic protease regulation

Substrate selection is not performed by the ClpP protease but rather by its partner ATPase. The diversity of these ATPases correlates with the diversity of substrates selected for proteolysis by ClpP. For example, *Bacillus subtilis* (though not a pathogenic bacterium) encodes the protease ClpP as well as three ATPases, which have been well-characterized: ClpX, ClpE, and ClpC (Figure 2A) (Gerth et al., 2004). These ATPases have all been shown to act on heat-damaged protein aggregates for subsequent ClpP-mediated turnover (Kruger et al., 1994; Derre et al., 1999; Kruger et al., 2000). Unlike ClpX, both ClpE and ClpC are under the transcriptional control of CtsR (class III stress *gene* repressor), which negatively regulates their expression (Derre et al., 1999; Kirstein and Turgay, 2005). ClpE is the most tightly repressed of the three ATPases and is only expressed under severe heat shock conditions (Miethke et al., 2006). Additionally, ClpE is further regulated through its turnover by ClpCP (Gerth et al., 2004).

As suggested above, a separation of functions is seen between the Clp ATPase chaperones. ClpX appears to be constitutively expressed and is available for maintenance of protein quality control at all times during the cell cycle, specifically for the removal of proteins whose translation has stalled (Flynn et al., 2001). Other ATPases are dedicated for degradation of substrates during stress, such as ClpC, which is the primary ATPase responsible for the degradation of non-native proteins (Kruger et al., 1994; Rouquette et al., 1996; Chatterjee et al., 2005). The presence of multiple substrate-selecting ATPases appears to be advantageous for the cell. Selective expression presumably allows the cell to diversify its use of the ClpP protease to meet the demands of the changes in the conditions imparted by a variety of environmental stresses.

In addition to the control resulting from encoding for multiple ATPases, cells possess the ability to modulate ClpP substrate selection co-translationally. One prominent example is the SsrA tagging system in prokaryotes, which is employed, for example, when truncated mRNAs are generated during aberrant or premature termination of transcription (Figure 2B) (Keiler et al., 1996). At the centre of this system is a transfer-messenger RNA (tmRNA), which is a tRNA that encodes for its own message; the message codes for a hydrophobic 8–35 residues long SsrA tag that is added to the C-terminus of incomplete nascent chains. During translation of truncated mRNAs, tmRNA binds to the A-site of a stalled ribosome and, subsequently, this leads to the translation of the complete SsrA tag that is added to the C-terminus of the nascent polypeptide (Keiler, 2015). In *Escherichia coli,* this tag is AANDENYALAA-(COO-) (Keiler et al., 1996), which is recognized by ClpX, leading to the degradation of the protein by ClpXP (Flynn et al., 2001; Farrell et al., 2007; Martin et al., 2008; Fei et al., 2020a). Proteins labelled with an SsrA tag can also be degraded by ClpAP, FtsH, and Lon (Gottesman et al., 1998; Herman et al., 1998; Choy et al., 2007).

The selectivity of ATPase chaperones is modulated by dedicated adaptors. Some adaptors are essential to ATPase function, such as in the case of ClpC in *B. subtilis,* where the binding of MecA, McsB, or YpbH adaptors (Schlothauer et al., 2003; Frees et al., 2007) is required for the assembly of an active hexameric chaperone (Kirstein et al., 2006). Recent data suggests that ClpC in *Staphylococcus aureus* and *B. subtilis* first assemble into a resting state comprised of a helical decamer; however, upon binding to an adaptor, the decamer reorganizes into an active hexameric state which can assemble with the ClpP tetradecamer (Carroni et al., 2017; Morreale et al., 2022). Other adaptors can modulate substrate selection but are not essential for ATPase function. This is true of ClpX that functions with several adaptor proteins. SspB (stringent starvation protein B) is one such adaptor that modulates the ClpX substrate pool by enhancing the degradation of SsrA tagged substrates (Levchenko et al., 2000; Dougan et al., 2003; Wah et al., 2003).

There is also diversity in the ClpP proteases themselves as shown in Figure 2A. The *E. coli* ClpP protease provides a baseline standard: a tetradecamer protein composed of two identical heptameric rings, where each heptameric ring of the active complex can associate with a Clp ATPase (Porankiewicz et al., 1999). However, several pathogenic organisms have two paralogs of ClpP encoded as: ClpP1 and ClpP2. For example, in *Pseudomonas aeruginosa*, ClpP1 can form a functional homotetradecamer protease, while ClpP2 cannot (Hall et al., 2017). Rather, ClpP2 assembles into a heptameric ring to associate with a ClpP1 heptamer. Thus, two active versions of the protease complex exist, ClpP1_14_ and ClpP1_7_P2_7_ (Figure 2A) (Hall et al., 2017). In *Mycobacterium tuberculosis,* ClpP1_14_ and ClpP2_14_ homotetradecamers can form but are inactive. It is only in the presence of activators that these subunits reassociate to form a ClpP1_7_P2_7_ heterotetradecamer capable of proteolysis (Akopian et al., 2012). Furthermore, the food-borne pathogen *Listeria monocytogenes* can form a functional ClpP2_14_ homotetradecamer and ClpP1_7_P2_7_ heterotetradecamer while the ClpP1_14_ is inactive (Zeiler et al., 2013) (Figure 2A). Additionally, in the anaerobic spore-forming gut bacterium *Clostridioides difficile*, both ClpP1_14_ and ClpP2_14_ homotetradecamers assemble into a functional protease (Lavey et al., 2019) (Figure 2A). Finally, in *Chlamydia trachomatis,* neither ClpP1 nor ClpP2 can form a homotetradecamer but rather associate with one another to create a ClpP1_7_P2_7_ heterotetradecamer capable of proteolytic activity (Pan et al., 2019) (Figure 2A). Currently, little is known about the advantages of producing multiple ClpP paralogs, however, ClpP complex asymmetry, and ATPase binding and peptidase specificity might provide additional levels of regulation for the protease (Personne et al., 2013; Leodolter et al., 2015; Nagpal et al., 2019).

## Role of caseinolytic protease in pathogen survivability

The pathogenicity of a bacterium is critically dependent on its ability to survive in a host. ClpP is known to influence many pathways involved in maintaining normal cellular functions. Classic ClpP trapping experiments have been used to identify substrates of ClpP (Flynn et al., 2003). In these experiments, ClpP^Trap^, an active site serine mutant of the protease that is unable to degrade proteins, is used. Substrates entering the ClpP^Trap^ will not be degraded but will be trapped inside the ClpP cylinder and are subsequently identified by pulling down ClpP^Trap^ followed by mass spectrometry analysis to identify trapped proteins. Using this approach in *S. aureus*, for example, central transcriptional and stress regulatory proteins were identified to be the main targets of the protease including Spx (global transcription regulator), CodY (transcriptional repressor) and FtsZ (essential Z-ring forming protein at site of cell division) (Michel et al., 2006; Frees et al., 2012; Feng et al., 2013; Panasenko et al., 2020) (Table 1). RecA (DNA damage repair protein), PerR (regulator of peroxide inducible genes), and DnaK (heat-shock protein 70) (Table 1) were also among the substrates of *S. aureus* ClpP, highlighting the key role ClpP plays in maintaining central biological functions such as DNA repair, cell division, and protein homeostasis (Krysiak et al., 2017; Kirsch et al., 2021). Additionally, the role of ClpP impacting pathogen survivability was seen in *M. tuberculosis,* since ClpP depletion led to a reduction in colony forming units both *in vitro* and in a mouse model (Raju et al., 2012b). One essential substrate of *M. tuberculosis* ClpP that was identified was Whib1 (Table 1), a transcriptional repressor whose lack of turnover and subsequent accumulation led to cell toxicity and thereby increased cell death (Raju et al., 2014).

**TABLE 1:** Bacterial proteins and virulence factors regulated by the ClpP system discussed in this review.

Pathway	Pathogen(s)	Protein(s)	Function	References
Cell growth and division	*S. aureus*	CodY	Transcriptional repressor	Frees et al. (2012)
	*S. aureus*	FtsZ	Z-ring forming protein at site of cell division	Michel et al. (2006)
	*S. aureus, S. epidermidis*	Spx	Global transcription regulator	Wang et al. (2010), Feng et al. (2013)
	*M. tuberculosis*	Whib1	Transcriptional repressor	Raju et al. (2014)
Stress regulation	*S. aureus*	DnaK	Heat shock chaperone	Kirsch et al. (2021)
	*S. aureus*	PerR	Regulator of peroxide inducible genes	Kirsch et al. (2021)
	*S. aureus*	RecA	DNA damage repair	Kirsch et al. (2021)
	*L. pneumophila, S. typhimurium*	CsrA	Global RNA binding protein	Martinez et al. (2011), Ge et al. (2019)
	*L. pneumophila*	IHFB	Transcriptional inhibitor of CsrA	Ge et al. (2019)
	*S. typhimurium, E. coli*	RpoS	General sigma S factor	Pratt and Silhavy (1996), Schweder et al. (1996), Iyoda and Watanabe (2005), Rice et al. (2015)
Peptidoglycan and biofilm synthesis	*S. aureus*	FemA, FemB, MurE, MurC, PBP2	Members of the peptidoglycan biosynthesis pathway	Feng et al. (2013)
	*P. aeruginosa*	AlgU	Sigma factor that initiates the transcription of genes involved in alginate production	Qiu et al. (2008)
	*P. aeruginosa*	MucA	Regulator of alginate production	Qiu et al. (2008)
Toxin-antitoxin system	*E. coli, S. aureus*	MazEF	MazE is an antitoxin that inhibits the mRNA- endoribonuclease toxin MazF	Donegan et al. (2010), Tripathi et al. (2014)
	*S. aureus*	TrfA	Adaptor that mediates MazE degradation by ClpCP	Panasenko et al. (2020)
	*E. coli*	ParDE	ParD is an antitoxin that inhibits the DNA gyrase inhibitor toxin ParE	Dubiel et al. (2018)
	*M. tuberculosis*	HigA1/HigB1	HigA1 is an antitoxin that inhibits the mRNA endoribonuclease toxin HigB1	Texier et al. (2021)
	*M. tuberculosis*	SecB-like chaperone	Chaperone that binds the C-terminal region of HigA1 and assists in its folding	Bordes et al. (2016)
	*M. tuberculosis*	VapB20/VapC20	VapB20 is an antitoxin that inhibits the 23 S rRNA endoribonuclease toxin VapC20	Winther et al. (2013)
	*M. tuberculosis*	RelB1/RelE1	RelB1 is an antitoxin that inhibits the mRNA endoribonuclease toxin RelE1	Korch et al. (2009)
	*E. coli*	GrlR	Regulator of T3SS genes expression	Iyoda et al. (2006)
Virulence factors and regulators	*L. monocytogenes*	Listeriolysin O	Hemolytic pore forming toxin	Gaillot et al. (2000)
	*S. aureus*	Rot	Repressor of toxins	Fisher et al. (2018)
	*S. aureus*	Hla	Pore-forming hemolysin alpha-toxin	Jenul and Horswill (2019)
	*S. aureus*	TSST	Toxic shock syndrome toxin	Ju et al. (2021)
	*S. aureus*	SEC	Enterotoxin C	Ju et al. (2021)
	*S. aureus*	SED	Enterotoxin D	Ju et al. (2021)
	*S. aureus*	MgrA	Autolytic activity regulator of the Agr quorum sensing pathway	Schelin et al. (2020)
	*S. pneumoniae*	Pneumolysin	Hemolytic pore forming toxin	Kwon et al. (2003)
	*S. pneumoniae*	PsaA	A virulence factor adhesin, also known as pneumococcal surface antigen A	Kwon et al. (2003)
Transport and motility	*Y. pestis*	LcrF	Transcriptional activator of the T3SS apparatus	Schwiesow et al. (2015)
	*Y. pestis*	YmoA	Regulator of expression of invasion proteins	Jackson et al. (2004)
	*E. coli, S. typhimurium*	FliC	Flagellin, a subunit of the flagellum filament	Tomoyasu et al. (2002), Iyoda et al. (2006)
	*E. coli, S. typhimurium*	FlhD/FlhC	Master regulators of flagellum biosynthesis	Tomoyasu et al. (2003), Kitagawa et al. (2011)

Below we discuss the role of ClpP and ClpX in pathways that are pertinent to a pathogen’s ability to survive in a host organism. Table 1 lists bacterial proteins that are discussed here, which are regulated by the ClpP system in different pathogens.

### Inhibiting caseinolytic protease disrupts peptidoglycan and biofilm formation

Maintaining the integrity of the cell wall is vital for bacterial survival, which is needed to prevent cytolysis when bacteria encounter the turgor pressure of the host cell’s cytoplasm. Loss of ClpXP activity has been linked to increased susceptibility to cell wall stress in *S. aureus*. It is thought that antibiotics targeting the cell wall result in protein damage and misfolding, and lead to increased production of chaperones and proteases to aid in protein turnover and repair (McGillivray et al., 2012; Michiels et al., 2016). Thus, inhibiting ClpX or ClpP likely hinders the clearance of such damaged proteins. This hypothesis is supported by observations in *S. aureus*, since Δ*clpP*, Δ*clpX*, or Δ*clpC* mutants showed an increase in peptidoglycan cross-linking and thicker cell walls (Baek et al., 2014). Indeed, a separate study purified ClpP^Trap^ from *S. aureus* and identified several enzymes involved in the peptidoglycan-synthesis pathway such as FemA, FemB, MurE, MurC, and PBP2 (Feng et al., 2013) (Table 1). While further research is required to better define the specific pathways modulated by ClpP, these results suggest that ClpP and its associated ATPases play an important role in maintaining the integrity of the cell wall.

In addition to peptidoglycan regulation, *clpP* deletion has been shown to dysregulate biofilm formation in *P. aeruginosa* (Qiu et al., 2008), *Staphylococcus epidermidis* (Wang et al., 2007) and *S. aureus* (Frees et al., 2004). For *P. aeruginosa,* overproduction of alginate, a central component of biofilms, leads to a mucoid phenotype, which indicates the onset of chronic lung infection in cystic fibrosis. A global genome-wide transposon mutagenesis screen for non-mucoid isolates of a mucoid strain led to the identification of ClpX, ClpP1, and ClpP2 as regulators of alginate production. Null mutants of *clpX, clpP1* or *clpP2* resulted in reduced alginate production in the non-mucoid strain, thereby implicating the role of Clp proteins in biofilm formation. It was found that ClpXP is required for the degradation of MucA, a negative regulator of the sigma factor AlgU (Table 1). By preventing the repression of AlgU by MucA, AlgU is available to bind the *algD* operon and initiate the transcription of genes involved in alginate production (Qiu et al., 2008).

Similarly, in an *S. epidermidis* Δ*clpP* strain*,* a decrease in virulence and biofilm formation was observed in a rat model of intravascular catheter-associated infection (Wang et al., 2007). Further investigation attributed this effect to increased levels of Spx, which also regulates exopolysaccharide production (Table1). It is thought that increased Spx levels leads to defects in the pathogen’s ability to initiate attachment, necessary for the early stages of biofilm establishment (Wang et al., 2010). Interestingly, in *S. aureus*, *clpP* deletion appears to enhance biofilm formation (Frees et al., 2004). However, *S. aureus* Δ*clpP* mutant CFU counts in a mouse model 7 days post infection were significantly reduced relative to wildtype *S. aureus,* suggesting that increased biofilm formation cannot compensate for the reduction in ClpP-mediated bacterial virulence and dissemination in the host (Liu et al., 2017).

### Inhibiting caseinolytic protease increases vulnerability to temperature

ClpP deletion appears to decrease the effectiveness of a pathogen’s innate stress responses against temperature changes. For example, in *Legionella pneumophila,* the causative agent of Legionnaires’ disease, Δ*clpP* mutants exhibited growth deficiency at 42°C, as well as reduced tolerance to heat shock treatment compared to the wild type bacterium (Li et al., 2010). These cells presented incomplete division and exhibited compromised colony formation. Similar findings were seen in a *Streptococcus pneumoniae clpP* knockout strain, where bacterial growth was severely impaired at 30°C, 37°C, and 43°C. Considering that this pathogen undergoes a drastic change in temperature as it enters the host bloodstream from the nasopharynx, it was thought that the survival of mice challenged with Δ*clpP S. pneumoniae* was ameliorated due to the role of ClpP in the heat shock response (Kwon et al., 2003; LaBreck et al., 2017). Furthermore, ClpP was also required for growth at 42°C in *Campylocabter jejuni,* a food-borne pathogen, whereby a Δ*clpP* mutant displayed an increase in the levels of misfolded protein aggregates (Cohn et al., 2007). Like *S. pneumoniae, C. jejuni* must maintain proper proteostasis to be able to overcome a drastic shift in temperatures during its life cycle to survive in the environment, as well as in avian carriers (42°C) and human hosts (37°C). Finally, in *Salmonella typhimurium,* the etiological agent of gastroenteritis, Δ*clpP* mutant growth was impaired in experiments subjecting the bacterium to high and low temperatures, high salt, and low pH conditions (Thomsen et al., 2002; Knudsen et al., 2014). Overall, the above observations appear to implicate the role of ClpP in removing misfolded proteins during abrupt temperature variations, thereby promoting bacterial virulence and resilience in the host.

### Inhibiting caseinolytic protease increases vulnerability to the host immune system

A common phenotype seen amongst pathogens that are deficient in protein homeostasis mechanisms is a lowered tolerance towards pH changes. Such pathogens are particularly susceptible to the phagocytic macrophages, which are part of the innate immune response. Upon the engulfment of a pathogen, macrophages acidify the phagosome and produce reactive oxygen species for bacterial killing (Westman and Grinstein, 2020). There are many studies that implicate the role of ClpP in the viability of intracellular pathogens following internalization by host cells. For instance, it was shown that *S. typhimurium* (Yamamoto et al., 2001), *L. monocytogenes* (Gaillot et al., 2000)*, L. pneumophila* (Zhao et al., 2016), *S. aureus* (Kim et al., 2020) and *S. pneumoniae* (Kwon et al., 2004) deleted of *clpP* exhibited impaired growth in macrophages, suggesting that ClpP is required for intracellular survival. Further studies on *S. pneumoniae* indicated that Δ*clpP* mutants exhibited increased sensitivity to oxidative stress and that treatment of macrophages by the nitric oxide synthase inhibitor S-methylisothiourea sulfate, led to a significant increase in Δ*clpP S. pneumoniae* viability (Lee et al., 2010). In addition, Δ*clpP S. pneumoniae* were found to stimulate apoptosis in dendritic cells less than their wildtype counterpart (Cao et al., 2013). Dendritic cells are antigen presenting cells that bridge the innate and adaptive immune responses together and are crucial for conferring long-term protection against pathogens (Banchereau et al., 2000). Thus, disruption of this network along with resistance against oxidative stress sheds light on the role of ClpP in promoting *S. pneumoniae* survival in the host. Interestingly, a similar observation was made for *S. aureus,* whereby a genome wide transposon screen revealed that Δ*clpP* strains showed a decrease in neutrophil lysis and that ClpP was necessary for growth and survival in a zebrafish embryo infection model (Yang et al., 2019). Therefore, ClpP seems to be implicated in modulating host cell immunity, thus disrupting the function of this protease in various intracellular pathogens confers protection against disease in the host.

### Caseinolytic protease regulates the type II toxin-antitoxin system

**FIGURE 3:**
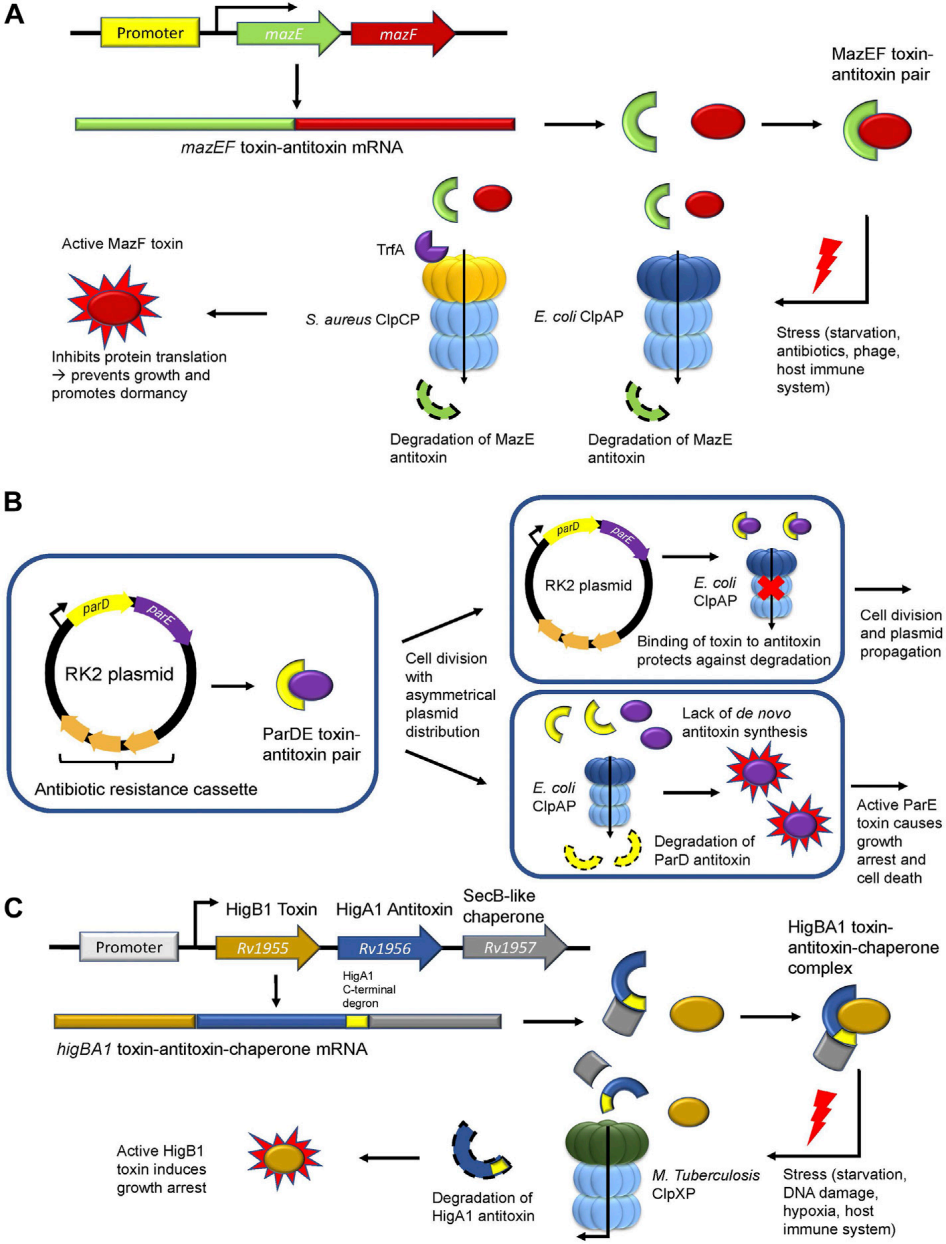
ClpP regulates the type II toxin-antitoxin system. **(A)** The *MazEF* toxin-antitoxin module. The MazE antitoxin (green) binds and inhibits the MazF toxin (red). During stress, ClpAP (*E. coli*) or ClpCP in association with TrfA (*S. aureus*) degrades the MazE antitoxin. Free MazF, which is an mRNA endoribonuclease, cleaves various intracellular targets leading to bacterial growth arrest. **(B)** The *parDE* toxin-antitoxin module. The RK2 plasmid encodes for the ParD antitoxin (yellow) and the ParE toxin (purple) and harbors an antibiotic resistance cassette (orange). Following asymmetrical cell division with unequal plasmid distribution to daughter cells, one of the progenies may not inherit any RK2 plasmid copies. Without the ability to synthesize the ParD antitoxin *de novo,* the antitoxin is degraded by ClpAP leading to free ParE toxin, which causes growth arrest. **(C)** The toxin-antitoxin-chaperone (TAC) module in *M. tuberculosis.* The SecB-like chaperone (grey) binds the C-terminal region (yellow) of the HigA1 antitoxin (blue) and assists in its folding and stabilization. HigA1 in turn binds and inhibits the HigB1 toxin (light brown). During stress, the SecB-like chaperone dissociates from HigA1 *via* an unknown signalling mechanism causing *M. tuberculosis* ClpXP to degrade the HigA1 antitoxin *via* the recognition of the now unmasked C-terminal degron. Free HigB1 toxin induces growth arrest causing the bacteria to enter a state of dormancy.

The bacterial toxin-antitoxin (TA) system consists of a constitutively active toxin and its cognate inhibitory, albeit labile antitoxin. TA modules are highly complex with eight systems characterized thus far (Types I-VIII) based on the composition of the antitoxin (RNA or protein) (Singh et al., 2021). During regular physiological conditions, the antitoxin is stable and inhibits the function of the toxin. However, during stress conditions, the antitoxin is degraded allowing the toxin to interact with effector proteins or DNA rendering a bacterial cell in a persistent dormant-like state. This is thought to assist the pathogen in resisting assault from antibiotic treatments, phage infections, and host immune defense mechanisms. (Harms et al., 2018; Singh et al., 2021). In type II TA modules, proteases such as ClpP stimulate this system by degrading the antitoxin, which causes the unrepressed toxin to inhibit translation and DNA replication, thereby promoting growth arrest (Page and Peti, 2016). One of the most well-documented type II TA modules is the MazEF system, which is comprised of the MazE antitoxin that inhibits the mRNA-cleaving endoribonuclease toxin MazF (Figure 3A; Table 1) (Zhang et al., 2003). In *E. coli,* ClpAP was shown to degrade MazE during starvation (Aizenman et al., 1996) to promote persister cell formation and survival following antibiotic treatment (Tripathi et al., 2014). In *S. aureus,* ClpCP degrades MazE (Donegan et al., 2010) but requires the adaptor TrfA to mediate this degradation (Figure 3A; Table 1) (Panasenko et al., 2020). Nevertheless, the downstream effects are the same as for *E. coli*, whereby the *mazEF* module in *S. aureus* is stimulated during oxidative and antibiotic stress to promote bacteriostasis (Fu et al., 2009; Panasenko et al., 2020). Deletion of either gene in the *mazEF* module or in the *clpCP* operon resulted in a significant reduction in persister cell formation and viability following antibiotic treatment (Schuster et al., 2015; Springer et al., 2016), demonstrating the role of ClpP-mediated proteostasis during stress.

The *parDE* system found in RK2 plasmids is another Type II TA module in *E. coli* that promotes bacterial persistence indirectly through plasmid maintenance in the bacterial population (Figure 3B). This system is comprised of the antitoxin ParD and the DNA gyrase inhibitor toxin ParE (Jiang et al., 2002) (Table 1). Following unequal cell division, a daughter cell may receive excess RK2 plasmids while the other daughter cell would be devoid of any RK2 plasmids. Given that these cells cannot synthesize the ParD antitoxin *de novo,* ClpAP degrades the remaining inherited ParD antitoxin, thereby causing ParE to inhibit DNA gyrase and promote growth arrest or cell death (Karlowicz et al., 2016; Dubiel et al., 2018). Maintenance of the RK2 plasmid ensures that the antibiotic resistance cassette encoding for kanamycin, ampicillin, and tetracycline found in this plasmid is retained in the population, thus enabling bacterial resistance against multi-drug treatments (Thomas et al., 1980; Villarroel et al., 1983).

Recently**,** the role of ClpXP in the regulation of the *rv1955-rv1957* TA system in *M. tuberculosis* was elucidated (Texier et al., 2021). The *rv1955-rv1957* operon encodes for an atypical toxin-antitoxin-chaperone (TAC) tripartite module consisting of HigB1 (toxin), HigA1 (antitoxin) and a SecB-like chaperone (Figure 3C; Table 1). The latter recognizes and binds a C-terminal region of the HigA1 antitoxin and assists in its folding (Bordes et al., 2016). In doing so, folded HigA1 antitoxin binds and inhibits the HigB1 toxin, preventing it from exerting its toxic effect. The C-terminal region also encompasses the degron sequence by which ClpXP recognizes HigA1 for degradation. Therefore, the binding of the chaperone to the C-terminal region of HigA1 masks the degron from recognition by ClpXP, thereby, inhibiting HigA1 turnover (Texier et al., 2021). However, following stress and through an unknown signalling mechanism, the SecB-like chaperone disengages from HigA1. Subsequently, ClpXP recognizes the C-terminal degron sequence on HigA1 and degrades it, thereby freeing HigB1 to exert its downstream toxic effect (Figure 3C) (Texier et al., 2021). The consequence of the HigB1 toxin activity includes growth inhibition (Schuessler et al., 2013). This TA module was shown to be upregulated in cell persisters (Keren et al., 2011), during starvation (Betts et al., 2002), DNA damage (Rand et al., 2003), hypoxia, and engulfment by phagocytes (Ramage et al., 2009), allowing the pathogen to withstand extreme environments. Such pathogens can become reactivated when conditions are favorable (Wayne and Sohaskey, 2001) and cause disease relapse, thereby highlighting the crucial role of this TA module during *M. tuberculosis* infection and its regulation by ClpXP.

In a recent proteomic screen and follow-up *in vitro* degradation assays, *M. tuberculosis* ClpCP was shown to degrade the VapB20 and RelB1 antitoxins, which bind and inhibit the VapC20 and RelE1 toxins, respectively (Ziemski et al., 2021) (Table 1). VapC20 enables growth arrest by cleavage of the Sarcin-ricin loop found on the 23 S ribosomal RNA, thereby, inhibiting protein translation (Winther et al., 2013). On the other hand, the RelE1 toxin is upregulated post-macrophage engulfment and nitrogen starvation (Korch et al., 2009; Korch et al., 2015). The activity of RelE1 compromises the structural integrity of the mycobacterial envelope and degrades mRNA, thereby, inhibiting protein translation and altering the proteome. Overall, ClpCP and ClpXP are essential in enabling cell persistence of *M. tuberculosis* by regulating the steady-state levels of antitoxins under a variety of environmental and biological stresses.

## The role of caseinolytic protease in modulating virulence and colonization

### caseinolytic protease influences the transition to virulent life cycle stages

ClpP plays an important role in facilitating the switch between the different life stages of certain bacteria. Pathogens such as *L. pneumophila* greatly rely on ClpP to transition between a replicative phase (RP) and an effector secreting virulent transmissive phase (TP) (Ge et al., 2022). One such regulator of this transition is CsrA (Table 1), whereby its accumulation in Δ*clpP L. pneumophila* mutants both inhibited the initiation of the non-virulent phase and reduced invasiveness to *Acanthamoeba castellanii* amoebae *in vitro.* Further investigation showed that the temporally expressed IHFB (Table 1), the transcriptional inhibitor of *csrA*, is degraded in a ClpP-dependent manner during the non-virulent phase. Thus, ClpP appears to be involved in regulating the transition both into and out of the virulent stage of *L. pneumophila* (Ge et al., 2019).

A similar switch between two life stages also occurs in the obligate intracellular pathogen *C. trachomatis*, which transitions between elementary bodies (EBs) and reticulate bodies (RBs)*.* EBs are the non-dividing infectious form, while RBs are the replicative non-infectious form (Elwell et al., 2016). It was found that transcripts of *clp* genes (*clpP1, clpP2, clpX, and clpC*) were greatly upregulated during the RB stage of the pathogen, suggesting that they play a role in cell cycle progression (Wood et al., 2019). A follow-up study showed that a Δ*clpP2* mutant disrupted the transition from the RB stage to the EB stage, while a Δ*clpX* mutant caused a morphological defect in EBs and decreased their viability (Wood et al., 2020).

Moreover, while no regulatory substrates were identified, ClpP was found to be required for sporulation in *C. difficile* (Bishop et al., 2022). The virulence and resilience of this pathogen to environmental stresses is critically dependent on spore formation (Shen, 2020). Spores formed from *C. difficile clpP1* and *clpP2* null mutants were found to be generally heat labile, morphologically abnormal, and they germinated significantly less than their wildtype counterpart (Bishop et al., 2022). Therefore, ClpP appears to be involved in regulating the transition between different life stages of several pathogens to modulate their virulence.

### Caseinolytic protease influences virulence factor expression

**FIGURE 4:**
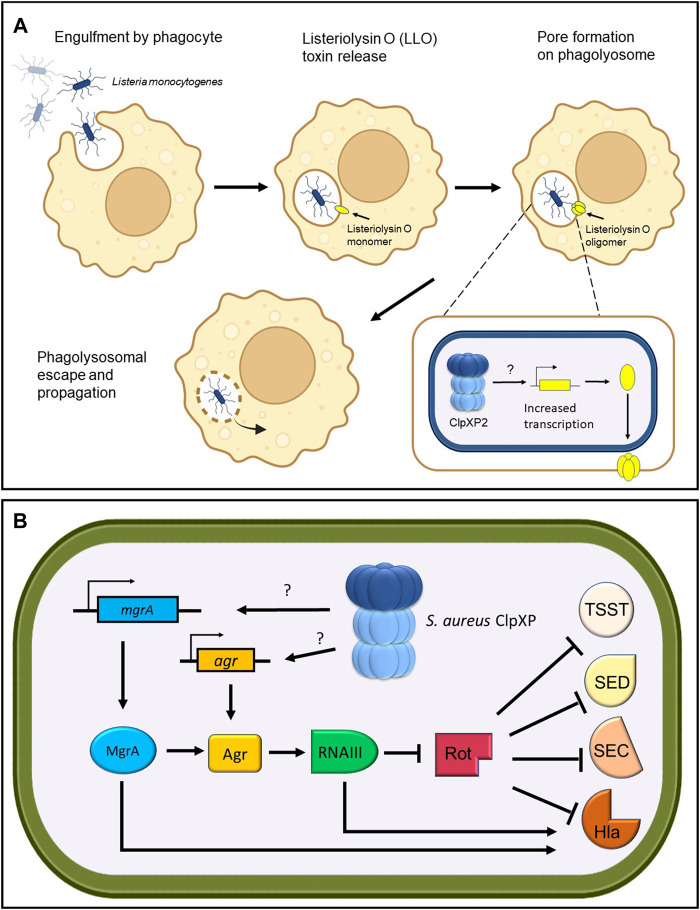
ClpP regulates toxin production. **(A)** *Listeria monoctyogenes* ClpXP2 transcriptionally increases the production of Listeriolysin O toxin for phagolysosomal escape and propagation. **(B)** *S. aureus* ClpXP regulates the production of toxic shock syndrome toxin (TSST), enterotoxin D (SED), enterotoxin C (SEC) and hemolysin alpha-toxin (Hla) through transcriptional control of the global master regulator *mgrA* and the *agr* quorum sensor. Through an unknown target, *S. aureus* ClpXP stimulates the transcription of *mgrA* and *agr* leading to RNAIII upregulation, which in turn inhibits the repressor of toxins (Rot). The inhibition of Rot enables the production of Tsst, Sed, Sec, and Hla toxins. In addition, MgrA and RNAIII can directly activate the production of Hla.

ClpP can affect virulence by regulating the transcription of specific virulence factors. In *L. monocytogenes*, it was found that ClpP2 is required specifically for the expression of Listeriolysin O (Table 1), a hemolytic toxin that forms pores in phagocytic vacuoles, allowing the pathogen to escape and propagate (Figure 4A) (Gaillot et al., 2000). Untangling these pathways into a linear “cause and effect” is a difficult task. However, it is likely that the reduced stress resistance in Δ*clpP2 L. monocytogenes* was the cause of the pathogen’s poor growth in mice, while reduced expression of Listeriolysin O resulted in its inability to propagate and cause infection. Similar transcriptional regulatory pathways have been shown to be modulated by ClpP in *S. pneumoniae*, whereby deletion of *clpP* reduced survival in infected mice (Kwon et al., 2003). Transcriptional analysis revealed that the Δ*clpP* mutants demonstrated decreased expression of the hemolytic pore-forming pneumolysin toxin and the adhesin pneumococcal surface antigen A (PsaA) (Table 1), suggesting that ClpP is a positive regulator of these virulence factors.

One of the most well-studied examples that implicates ClpP within a complex regulatory network that controls virulence factor expression stems from extensive studies on *S. aureus.* The Agr quorum sensor in *S. aureus* controls the expression of virulence factors ultimately through the transcription of RNAIII, a regulatory RNA effector of the *agr* regulon (Figure 4B). RNAIII in turn blocks the expression of the repressor of toxins (Rot) and stimulates production of toxins such as the pore-forming hemolysin alpha-toxin (Hla) (Table 1). Repression of *rot* also allows the transcription of virulence factor genes such as toxic shock syndrome toxin (*tsst*), enterotoxin C (*sec*) and enterotoxin D (*sed*) (Fisher et al., 2018; Jenul and Horswill, 2019; Ju et al., 2021) (Table 1). *S. aureus* Δ*clpXP* mutants showed attenuated virulence in a murine skin abscess model, whereby hemolysin alpha-toxin protein and expression levels were reduced concomitant with a reduction in *agr* and RNAIII transcripts (Frees et al., 2003; Stahlhut et al., 2017). Therefore, this suggests that ClpXP positively regulates *hla* expression through the *agr* locus (Frees et al., 2003). Interestingly, in *S. aureus clpXP* null mutants, expression of *tsst, sed* and sec were not affected despite the reduction in Rot protein levels. However, the global master regulator *mgrA* was downregulated (Schelin et al., 2020) (Table 1). MgrA is known to modulate the activity of more than 350 genes (Luong et al., 2006) and can stimulate Hla production in a dual fashion-*agr* dependent and *agr* independent (Ingavale et al., 2005). Therefore, this implies that *tsst, sed,* and *sec* expression might be postively regulated by ClpXP through the upregulation of *mgrA*, but further investigation is required.

### Caseinolytic protease influences virulence factor transport systems

Virulence factors can be secreted into the extracellular environment or directly into host cells. Typically, this is accomplished through specialized transport and secretion systems or both. These secretion systems (Types I-VII) use a single energy-coupled step to transport proteins across membranes and are important for injecting bacterial virulence factors out into the extracellular environment or directly into host cells, causing a signal transduction cascade. The consequence of this may include disruption of essential signalling pathways and cytoskeletal remodelling (Asrat et al., 2015; Green and Mecsas, 2016). Expression and turnover of these bacterial secretion systems are regulated by ClpP in several pathogens. Recently, *clpP* deletion in *L. pneumophila* was shown to downregulate and upregulate an extensive array of type IVB secretion system and effector proteins. These proteins were differentially expressed depending on the life cycle stage of the bacterium (replicative or transmissive). Notably, of the 428 differentially expressed proteins, 316 were found to be modulated in a ClpP-dependent manner suggesting that ClpP plays a major role in manipulating host cell machinery through these secretion systems and effector proteins (Ge et al., 2022).

**FIGURE 5:**
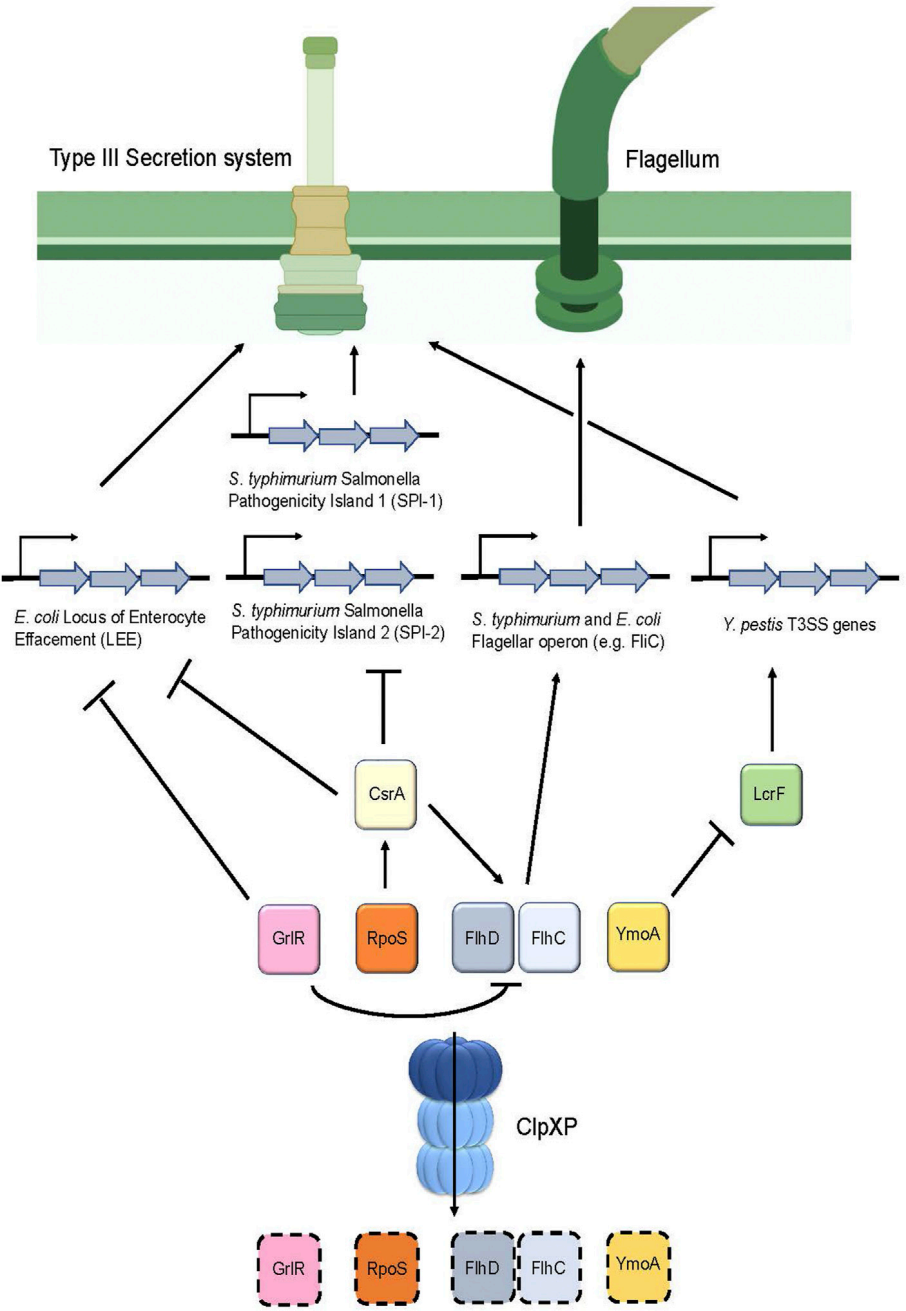
ClpP regulates the assembly of the Type III secretion system as well as flagellum synthesis in various pathogens. Degradation of the repressor GrlR by ClpXP removes the inhibition of transcription of the Locus of the Enterocyte Effacement (LEE) genes, which encode for the T3SS apparatus in *E. coli*. In addition, degradation of GrlR prevents repression of FlhD and FlhC, the master regulators of flagellar genes, thereby causing flagellum synthesis to occur. Furthermore, RpoS turnover by ClpXP prevents upregulation of CsrA, thereby causing expression of LEE in *E. coli* and *Salmonella* Pathogenicity Islands 1 and 2 in *S. typhimurium.* Additionally, CsrA positively regulates FlhD and FlhC expression, therefore, turnover of RpoS by ClpXP ultimately results in FlhD and FlhC downregulation through CsrA, leading to decreased flagellar synthesis. In *Y. pestis,* degradation of the repressor YmoA enables LcrF to stimulate production of the T3SS apparatus.

Additionally, ClpP appears to directly regulate Type III secretion system (T3SS) genes in *Yersinia pestis* (Jackson et al., 2004) (Figure 5). In this pathogen, expression of the T3SS apparatus is mediated by the transcriptional activator LcrF and is repressed by a histone-like protein termed YmoA (Schwiesow et al., 2015) (Table 1). Deletion of *clpP* in *Y. pestis* caused the pathogen to express a non-functional T3SS, which was revealed to be, in part, due to the upregulation of YmoA. Therefore, this suggests that YmoA is a target of ClpXP, and that its proteolytic degradation enables LcrF to express the genes found in the T3SS apparatus (Figure 5) (Jackson et al., 2004).

A similar mechanism involving the regulation of the T3SS by ClpP was found in *S. typhimurium* (Figure 5)*.* This bacterium possesses two distinct T3SS apparatuses that are encoded by two separate *Salmonella* pathogenicity islands (SPI-1 and SPI-2) for invasion and pathogenesis, respectively (Pizarro-Cerda and Cossart, 2006). There are several regulators that control these islands; noteworthy are the two negative regulators: the sigma factor RpoS (Rice et al., 2015) and the global RNA binding protein CsrA (Martinez et al., 2011) (Table 1). *ClpP* deletion in *S. typhimurium* showed that *rpoS* and *csrA* mRNA expression were upregulated. Further, *csrA* mRNA expression was reduced in the *rpoS* and *clpP/rpoS* double deletion mutants, suggesting that *csrA* is modulated by RpoS (Knudsen et al., 2013). Based on these results, and the observation that RpoS is a substrate of ClpXP in *E. coli*, (Pratt and Silhavy, 1996; Schweder et al., 1996), it is thought that ClpP in *S. typhimurium* positively controls the expression of the T3SS by regulating *csrA* levels indirectly through RpoS turnover (Figure 5).

In *E. coli,* attaching and effacing lesions in the intestinal mucosa produced by this pathogen are encoded on a pathogenicity island termed the locus of enterocyte effacement (LEE). These genes encode for chaperones, structural and effector proteins that comprise the *E. coli* T3SS (Furniss and Clements, 2018). LEE appears to be positively regulated by ClpXP as evidenced by a significant reduction of the LEE-encoded Esp proteins following *clpXP* deletion. Further analysis indicated that ClpXP modulates the expression of LEE *via* two distinct mechanisms (Figure 5). First, deletion of the LEE negative regulator *grlR* showed a comparable increase of *esp* expression in both the Δ*grlR* mutants and in the Δ*grlR/*Δ*clpXP* mutants, indicating that *grlR* expression is under the control of ClpXP (Iyoda and Watanabe, 2005). Second, deletion of the sigma factor *rpoS,* a known substrate of ClpXP (Schweder et al., 1996) in the *clpXP* mutants, resulted in a partial increase in *esp* expression (Iyoda and Watanabe, 2005). *rpoS* overexpression through insertion of a multicopy plasmid in these Δ*rpoS/*Δ*clpXP* mutants significantly repressed *esp* expression. Taken together, these results suggest that ClpXP positively modulates LEE expression through RpoS and GrlR turnover (Figure 5; Table 1).

### Caseinolytic protease influences motility

Bacterial motility is essential for pathogens to navigate through the environment to obtain nutrients and to seek a host for infection and colonization. Motility strongly contributes to the disease-causing capabilities of a pathogen, with those lacking such mechanisms exhibiting attenuated virulence (Josenhans and Suerbaum, 2002). Given that the T3SS injectisome largely resembles the supramolecular structure of the flagellar complex, the T3SS is likely derived from a flagellar common ancestor (Diepold and Armitage, 2015). It is postulated that ClpXP regulates motility by modulating flagellum synthesis. Indeed, *clpXP* deletions in *S. typhimurium,* resulted in cells exhibiting a “hyperflagellated” phenotype characterized by hypermobility and an increased rate of *fliC* transcription that encodes for flagellin, a subunit of the flagellum filament (Tomoyasu et al., 2002) (Figure 5; Table 1). Further analysis showed that *clpXP* deletions increased and stabilized the half-lives of the FlhD/FlhC proteins, which are the master regulators that encode for all the genes in the flagellum complex. Therefore, ClpXP acts as a negative regulator of flagellum synthesis by post-translationally regulating FlhD/FlhC turnover (Tomoyasu et al., 2003) (Figure 5; Table 1). The role of ClpXP in regulating flagellar synthesis was also reported in *E. coli*. ClpXP deletion led to an upregulation of *fliC* expression (Iyoda et al., 2006), as well as FlhD/FlhC accumulation and stabilization (Kitagawa et al., 2011). Moreover, deletion of *grlR* led to the downregulation of *flhD* transcripts and, correspondingly, *fliC* expression (Iyoda et al., 2006). Given that ClpXP was also shown to downregulate GrlR protein levels (Iyoda and Watanabe, 2005), this suggests that ClpXP negatively regulates flagellar synthesis directly through FlhD/FlhC and GrlR turnover in *E. coli* (Figure 5).

Currently there is a lack of mechanistic studies implicating ClpXP in *P. aeruginosa* motility. Nevertheless, phenotypic effects were observed upon *clpXP* deletion whereby twitching, swimming and swarming motility were impaired (Shanks et al., 2006; Fernandez et al., 2012). Importantly, ClpP1 but not ClpP2 is required for both swimming and twitching motility (Hall et al., 2017). Swimming motility refers to the individual movement of bacteria, whereas swarming motility refers to bacteria that migrate en masse; both types mediated by flagellar rotation. In twitching motility, bacteria migrate on surfaces through attachment and retraction of the pili appendages (Kearns, 2010). Overall, ClpXP appears to be involved in the regulation of flagellar and pilus synthesis in *P. aeruginosa*, but further studies are needed to elucidate signalling pathways and regulatory mechanisms that are at play.

## Caseinolytic protease as an antibiotic target

In the previous sections, the role of ClpP and its cognate ATPases in pathogenesis was highlighted, demonstrating that the protease not only influences a pathogen’s survivability, but also its infectivity potential and virulence. As such, much research has gone into the discovery and optimization of compounds that can interfere with the regulatory roles of ClpP and its associating ATPases.

Chemical alteration of ClpP activity has been shown to affect the virulence and infectivity of several pathogens (Conlon et al., 2013; Brӧtz-Oesterhelt and Sass, 2014; Silber et al., 2020). One strategy employed is the use of compounds such as β-lactones/lactams, phenyl esters, peptide boronic acids, and clipibicyclene as ClpP inhibitors (Gersch et al., 2013; Akopian et al., 2015; Hackl et al., 2015; Culp et al., 2022). These compounds typically bind to the active site serine of ClpP to prevent proteolysis. The mechanism of action of β-lactones/lactams, phenyl esters and peptide boronic acids on ClpP were comprehensively discussed in these reviews from our group and others (Culp and Wright, 2017; Bhandari et al., 2018; Moreno-Cinos et al., 2019; Mabanglo and Houry, 2022).

The unique structure and regulation of the ATPase-ClpP complexes allows for an additional mode of chemical interference of ClpP activity. Acyldepsipeptides (ADEPs) and activators of self-compartmentalizing proteases (ACPs) have been shown to bind the hydrophobic pockets of ClpP that are normally occupied by the ATPase’s IGF loops (Brӧtz-Oesterhelt et al., 2005; Martin et al., 2007; Lee et al., 2010; Leung et al., 2011; Goodreid et al., 2014; Goodreid et al., 2016; Mabanglo et al., 2019; Binepal et al., 2020) (Figure 1B). This binding results in the widening of the tetradecameric ClpP axial pores, which facilitates substrate entry and increases proteolytic activity (Li et al., 2010a). Association of the drugs to ClpP disrupts the chaperone-ClpP interaction while keeping ClpP in its active conformation, resulting in unregulated proteolysis (Mroue et al., 2019; Jacques et al., 2020). These compounds have been shown to induce promiscuous degradation of non-substrate proteins both *in vitro* and *in vivo*, thereby resulting in cell death (Kirstein et al., 2009; Li et al., 2010a) and validating ClpP as a promising antimicrobial target. It is worth noting that in firmicutes (e.g., *Bacillus, Staphylococcus,* and *Streptococcus*), ClpP is not essential for cell viability. The bactericidal activity of ADEPs is mainly due to unregulated proteolysis of substrates by ClpP such as FtsZ, which is required for cell division (Sass et al., 2011). However in *M. tuberculosis, clpP1* deletion leads to cell death making it an essential component of the proteostasis network (Ollinger et al., 2012); accordingly, the mechanism by which ADEPs induce cell death in mycobacteria is different. While ADEPs can stimulate degradation of peptides and full-length proteins in *M. tuberculosis,* its effect is weaker than in firmicutes and other bacteria. Rather, the binding of ADEPs inhibits the association of ClpP1P2 with the ATPases ClpX or ClpC1 to prevent essential protein turnover in mycobacteria, thus, leading to cell death (Famulla et al., 2016).

It may seem like a negative trait for an antibacterial target to possess multiple paralogs and regulatory pathways that can compensate for each other. However, this can be advantageous because disruption of the ClpP-chaperone complex’s activity can increase the bacterium’s susceptibility to the host’s immune system and to other antibiotics without imposing strong evolutionary pressure, which gives rise to resistant mutants. Indeed, co-administering traditional antibiotics with ClpP dysregulators has been shown to be effective at killing otherwise resistant mutants. Incubating high density cultures of methicillin resistant *Staphylococcus aureus* (MRSA) and vancomycin-resistant *Enterococcus faecalis* (VRE) with linezolid, ampicillin, oxacillin, or ADEPs individually, showed that none of the compounds had bactericidal activity against either of the two species. However, co-incubation of linezolid, ampicillin, or oxacillin with ADEPs was found to be effective at killing and preventing regrowth of the pathogens, with no evidence of developing resistant mutants (Mroue et al., 2019).

However, targeting ClpP function as an effective anti-virulence strategy with small molecule compounds has some caveats. In addition to the above-mentioned *S. aureus* infection model, a study treating *L. monocytogenes* with β-lactones in mouse macrophages showed a significant reduction in intracellular replication. Follow up analyses confirmed that this was due to the compound binding to ClpP (Bottcher and Sieber, 2009). Here, disruption of ClpP function was seen to decrease virulence. In contrast, deletion of *clpX* and *clpP* from MRSA led to increased resistance to β-lactam antibiotics (Baek et al., 2014). These results suggested that ClpXP controls one or more of the pathways modulating β-lactam resistance in *S. aureus*. MRSA expresses a peptidoglycan transpeptidase, penicillin binding protein 2a (PBP2a), which has decreased affinity for β-lactams compared to other PBPs enabling resistance against β-lactam antibiotics (Baek et al., 2014). In the *clpXP* knockout mutants, cellular levels of PBP2a were found to be unchanged suggesting that PBP2a mediated resistance against β-lactam antibiotics is still intact. Nevertheless, it was noted that these mutants possessed generally thicker cell walls consisting of an altered muropeptide composition and increased levels of crosslinking (Baek et al., 2014). Therefore, it is likely that the role of ClpXP in the peptidoglycan synthesis pathway is multifaceted, which led to this undesirable phenotype (Baek et al., 2014).

Discrepancies in drug effectiveness like the ones described above may also in part be due to the various functional forms of ClpPs found within different bacterial species. Specific ClpP paralogs are known to serve different functions, and the expression levels of each paralog have been shown to fluctuate through various growth stages for some pathogens. For example, in *P. aeruginosa, clpP1* and *clpX* were observed to be constitutively expressed throughout the cell growth cycle, while *clpP2* expression was shown to increase during the stationary phase (Hall et al., 2017). Similar differences between paralogs have been recorded for other species such as *L. monocytogenes* and *C. difficile* (Balogh et al., 2017; Lavey et al., 2019; Mawla et al., 2021). Furthermore, variations in encoded ATPases could contribute to these discrepancies by minimizing the effects of dysregulating any specific chaperone. The redundancy of ClpC and ClpE suggests that many of the pathways dysregulated by inhibiting ClpC could be compensated for by the presence of ClpE except in *M. tuberculosis,* which lacks the latter Clp ATPase. ClpC1 dysregulators in *M. tuberculosis* such as ecumicin and lassomycin possess bactericidal properties by enhancing the ATPase activity of ClpC1 and uncoupling its proteolytic activity from ClpP1P2 (Gavrish et al., 2014; Gao et al., 2015). By dysregulating proteostasis and causing cell death*,* ClpC1 appears to be a viable drug target against *M. tuberculosis.* Nevertheless, how the overlapping functions of multiple paralogs or chaperones factor into the efficacy of antibiotics in other species is not well understood. Further research is needed to design and administer effective antibiotics that are specific towards particular pathogens, accounting for variations in ClpP and chaperones.

An additional consideration with respect to drug design is the presence of a ClpP in the mitochondrion of eukaryotes. Novel antibiotic inhibitors and activators therefore must be highly specific for prokaryotic ClpP, so as not to target human ClpP. Indeed, it has been shown that mitochondrial ClpP is also dysregulated by ADEPs (Lowth et al., 2012; Wong et al., 2018).

## Concluding remarks

In summary, ClpP and its cognate ATPases affect bacterial virulence through its involvement in peptidoglycan and biofilm formation, cell stress tolerance, motility, defense against host immune responses, the shift to a virulent life stage, and the production and transport of virulence factors. Although the presence of multiple ClpPs and the redundancy of Clp chaperones suggest that the protease is not essential for pathogen survival, chemical compounds that dysregulate ClpP have been shown to induce anti-virulence effects and increase bacterial susceptibility towards other antibiotics. It is worth noting that most contemporary antimicrobial compounds targeting ClpP were tested using *S. aureus* or *E. coli*, necessitating further investigation into other pathogens as well. Nevertheless, ClpP is a desirable druggable target and future research should be aimed at optimizing ClpP antimicrobial compounds in order to validate their use in animal models and eventually humans.
